# Inflammasomes and their roles in arthritic disease pathogenesis

**DOI:** 10.3389/fmolb.2022.1027917

**Published:** 2022-10-28

**Authors:** Gabsik Yang, Han Chang Kang, Yong-Yeon Cho, Hye Suk Lee, Joo Young Lee

**Affiliations:** ^1^ Department of Pharmacology, College of Korean Medicine, Woosuk University, Jeonju, South Korea; ^2^ College of Pharmacy, The Catholic University of Korea, Seoul, South Korea

**Keywords:** inflammation, pharmacological inhibitors, therapeutic targets, immunity, pattern recognition receptors

## Abstract

The inflammasome is a molecular platform that is created in the cytosolic compartment to mediate the host immunological response to cellular injury and infection. Caspase-1 may be activated by the inflammasome, which leads to the generation of the inflammatory cytokines interleukin-1β (IL-1β) and IL-18 and the beginning of pyroptosis, which is a type of proinflammatory cell death. Scientists have identified a number of different inflammasomes in the last 2 decades. The NLRP3 inflammasome has been studied the most, and its activity may be triggered by a broad range of different inducers. However, activation of the NLRP3 inflammasome in a manner that is not properly controlled is also a factor in the etiology of many human illnesses. Accumulating evidence indicates that the NLRP3 inflammasome plays a significant role in the innate and adaptive immune systems and the development of various arthritic illnesses, such as rheumatoid arthritis, ankylosing spondylitis, and gout. The present review provides a concise summary of the biological properties of the NLRP3 inflammasome and presents the fundamental processes behind its activation and control. We discuss the role of the inflammasome in the pathogenesis of arthritic diseases, such as rheumatoid arthritis, ankylosing spondylitis, and gout, and the potential of newly developed therapies that specifically target the inflammasome or its products for the treatment of inflammatory diseases, with a particular emphasis on treatment and clinical application.

## Introduction

Autoimmune disorders, such as rheumatoid arthritis (RA), gout, systemic lupus erythematosus (SLE), ankylosing spondylitis (AS), juvenile idiopathic spinal arthritis, and Sjögren’s syndrome (SS), are characterized by the loss of immunological tolerance to autoantigens and persistent autoresponsive immune responses, which culminate in the overproduction of autoantibodies and organ damage (Zhernakova et al., 2013). However, the precise process of autoimmune disease initiation is not clear, which makes the treatment of these conditions challenging.

Inflammation is a congenital immune response that is primarily caused by macrophages (myeloid immune cells) and initiated by pattern recognition receptors (PRRs) recognition of pathogen-associated molecular patterns (PAMPs) or danger-associated molecular patterns (DAMPs) induced by infection or intrinsic stress (Janeway and Medzhitov 2002). PRRs include receptors that are present on the cell membrane, such as Toll-like receptors (TLRs), that are present in the cytoplasm, such as nucleotide-binding domain-like receptors (NLRs) and inflammasomes (Schroder et al., 2010). Inflammasomes are protein complexes comprised of a receptor protein, an adaptor, and procaspase-1. These are “canonical” inflammasomes, with NLRs and absent in melanoma 2 (AIM2) as a receptor protein and apoptosis-associated speck-like protein containing a caspase-recruitment domain (ASC) as an adaptor protein. Formation of inflammasomes is induced upon a variety of stimuli, including PAMPs derived from infection and DAMPs derived from host damage, which culminate in the maturation of pro-inflammatory cytokines, such as interleukin (IL)-1β and IL-18, that activate host immunity. A “non-canonical” inflammasome pathway leads to activation of caspase-4 and -5 in humans and caspase-11 in mice in response to intracellular LPS. The NLR family has at least 22 members, including NLRP1, NLRP3, and NLRC4, with a central nucleotide binding and oligomerization (NACHT) domain, caspase recruitment (CARD) domain, and leucine-rich repeats (LRRs) or a pyrin (PYD) domain. The NLRP1 inflammatory complex is a protein complex consisting of NLRP1, ASC, and pro-caspase-1 (Lamkanfi and Dixit 2014), was first identified in the NLR family. The pro-inflammatory cytokines pro-IL-1β and pro-IL-18 are cleaved to release the active cytokines IL-1β and IL-18, respectively (Broz and Dixit 2016). The NLRP3 inflammasome is predominantly expressed in peripheral blood leukocytes, and an NLRP3 inflammatory complex coupled with ASC and pro-caspase-1 rapidly forms in response to inflammatory stimuli. Formation of the NLRP3 inflammatory complex activates caspase-1, which leads to maturation of the pro-inflammatory cytokines IL-1β and IL-18 (Seok et al., 2020). IL-1β plays a multifunctional role in the immune response to induce cytokine production, enhance T-cell activation and antigen recognition, guide innate immune cells to the site of infection (Dinarello 2011; van de Veerdonk and Netea 2013), and induce activation of the NF-κB signaling cascade to induce cytokine production, which results in the transcriptional activation of genes encoding chemokines and various pro-inflammatory mediators (Kanneganti et al., 2007). The NLRP3 inflammasome is linked with the sensitivity, disease severity, and treatment outcomes of autoimmune disorders (Seok et al., 2021). This review examines the function of the NLRP3 inflammasome in the development of autoimmune disorders, such as RA, AS, and gout, based on previous studies and highlights current research on the involvement of inflammasomes in inflammatory and rheumatic autoimmune disorders.

## The pathology of rheumatic autoimmune disorders

Rheumatoid arthritis (RA) is an autoimmune disease that is characterized by chronic inflammation and joint pain. RA involves the inflammation of active joints, cartilage destruction, bone erosion, and calcification around the joints, which result in joint dysfunction. RA is one of the most common inflammatory autoimmune disorders worldwide (George et al., 2020), and 1%–2% of the world’s population suffers from RA. Its incidence is increasing 8.2% annually (Crowson et al., 2011; Safiri et al., 2019). Autoimmunity is the first step in RA pathogenesis, and high serum concentrations of autoantibodies, such as anticitrullineated peptide antibodies (ACPAs), are hallmarks of RA, although some patients are seronegative for RA (Firestein 2003; McInnes and Schett 2011). The exact pathogenesis of RA is not clear, but genetics, smoking, obesity, infection, periodontal disease, and the gut microbiota are likely involved (Muruve et al., 2008).

Ankylosing spondylitis (AS) is a chronic inflammatory disease that primarily causes inflammation and new bone formation in the axial skeleton and peripheral joints (Dougados and Baeten 2011). AS is a type of spondyloarthritis (SpA), and the pathological feature of SpA is the occurrence of ankylosing in the axial joint (Maksymowych et al., 2012). Maksymowych divided the symptoms of AS into inflammation, destruction, recovery, and extra-articular features (Maksymowych 2010): 1) inflammatory features of peripheral synovitis and adhesions; 2) destructive characteristics, such as loss of cartilage and bone; 3) restorative repair characteristics of new bone formation; and 4) inflammatory bowel disease (IBD), acute anterior encephalitis (AAU), and extra-articular features, such as psoriasis. Inflammation, which is the main initial symptom of AS, gradually progresses to bone abnormality (bone loss), joint stiffness, and joint fusion, which result in ankylosing of the spine (Quaden et al., 2016). Whether there is a correlation between bone formation and inflammation, which is one of the main features of AS, remains controversial (Maksymowych et al., 2012). However, the mechanism and pathogenesis of AS are not clear despite active research. AS is a multifactorial disease that is affected by genetic factors, such as HLA-B27, environmental factors, such as intestinal bacteria, and immunological factors (Tsui et al., 2014). Epidemiological studies showed that AS primarily occurred in men their twenties and forties (Zhu et al., 2019), with a worldwide prevalence of 0.1%–1.4% (Akkoc 2008).

Gout is the most common form of inflammatory arthritis (Stamp and Farquhar 2021), and it is caused by the accumulation of monosodium urate (MSU) crystals in joints, tendons, and other tissues due to elevated serum urate levels (Dehlin et al., 2020). According to recent data from the National Institute of Health and Nutrition (NIHN), the prevalence of gout is generally higher in men (5.2%) than women (2.7%) (Chen-Xu et al., 2019), and the risk of developing gout increases with age (Dehlin et al., 2020). A tophus is a granuloma that histologically surrounds urate crystals and tissue debris (Schweyer et al., 2000). Acute gout attacks are caused by the body’s immune response to tophi (Lee, 2011).

## Rheumatoid arthritis and the inflammasome

Genetic factors play a pivotal role in RA pathogenesis, and several genes contribute to disease induction (Crowson et al., 2011; George et al., 2020). Most of the genetic mutations related to RA development are related to the immune response, and mutations in human leukocyte antigen (HLA) and the inflammatory complex are major genetic risk factors (Crowson et al., 2011). Variations in the *NLRP1* gene increase NLRP1 expression, which is a risk factor for the development of inflammatory diseases, including RA, type 1 intrinsic diabetes, vitiligo, and associated autoimmune diseases (Jin et al., 2007; McInnes and Schett 2011; Sui et al., 2012; Levandowski et al., 2013). Mutations in the *NLRP3* gene play an important role in several diseases, including gouty arthritis and type I diabetes (Pontillo et al., 2010; Zhang et al., 2018), and the expression of NLRP3 inflammatory complex-related genes (*NLRP3*, *ASC*, and *CASP1*) in peripheral blood mononuclear cells (PBMCs) may play an important role in RA pathogenesis and disease activity (Cheng et al., 2021). Overactivation of the NLRP3 inflammatory complex leads to excessive inflammation and unnecessary host tissue damage, which contribute to the severity of RA (He et al., 2016). The activity and expression of NLRP3 are upregulated in RA patients, which further suggests that the NLRP3 inflammatory complex may play an essential role in RA pathogenesis (Mathews et al., 2014; Choulaki et al., 2015; Ruscitti et al., 2015; Kim H et al., 2017; Addobbati et al., 2018). The expression of NLRC4 was markedly higher in monocytes derived from Brazilian RA patients (Addobbati et al., 2018).

**FIGURE 1:**
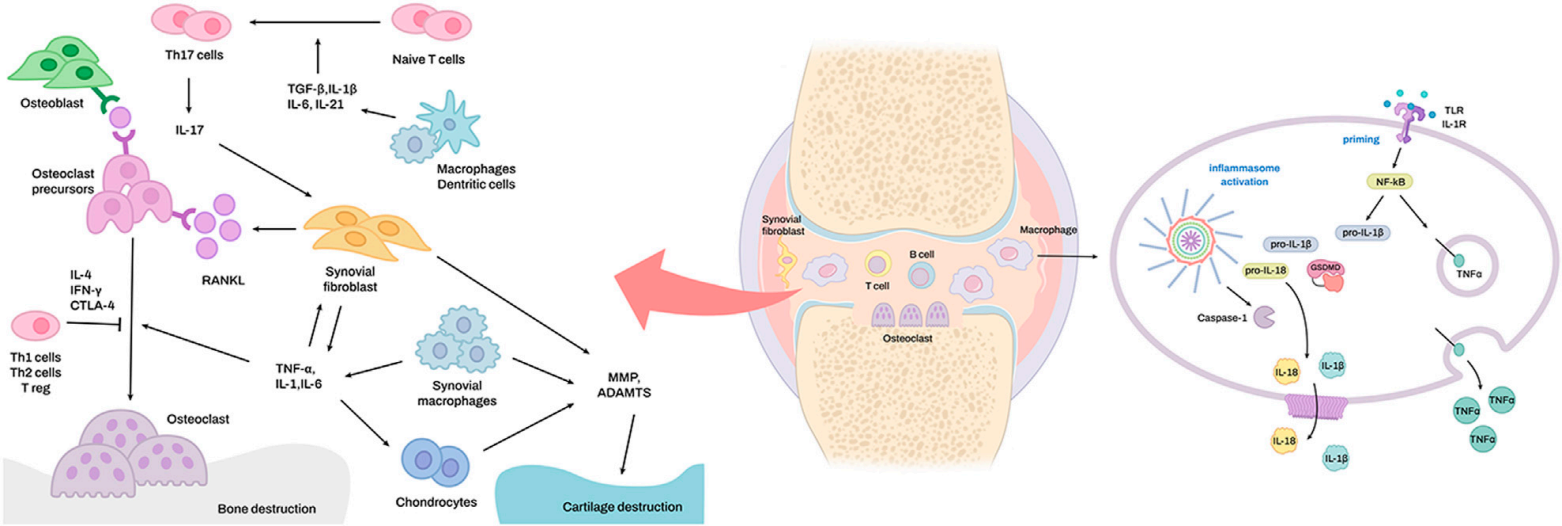
The signaling pathways for inflammasome activation in rheumatoid arthritis. Overexpression of TNF-α in the synovial macrophages of rheumatoid arthritis joints leads to activation of the classical NF-κB pathway, which leads to activation of the transcription factor RelA. RelA constructively regulates the expression of NLRP3. In conclusion, a higher level of NLRP3 expression results in more robust activation of the NLRP3 inflammasome.

Cartilage decomposition in RA occurs due to an excessive immune response (Komatsu and Takayanagi, 2022). Pro-inflammatory cytokines, such as IL-1, tumor necrosis factor (TNF), IL-6, and IL-17, induce cartilage-degrading enzymes, such as matrix metalloproteinases (MMPs) and aggrecanases (e.g., ADAM metallopeptidase with thrombospondin type 1 motif [ADAMTS]-4 and ADAMTS-5). These factors induce the production of synovial fluid and inhibit extracellular matrix production by chondrocytes in synovial fibroblasts (Pap and Korb-Pap, 2015; Araki and Mimura, 2017). This inflammatory reaction primarily occurs *via* the formation of an inflammatory complex called an inflammasome, which results in the formation and secretion of inflammasome-specific cytokines, such as IL-1β and IL-18, in macrophages and the inflammatory necrosis of macrophages called pyroptosis (Figure 1) (Lamkanfi and Dixit, 2014; Broz and Dixit, 2016). Pro-inflammatory cytokines, such as TNF, IL-6, and IL-1, which are abundant in the synovial membrane and synovial fluid of patients with RA, promote RANKL expression by synovial fibroblasts, and TNF and M-CSF induce osteoclast production (Takayanagi, 2007; Okamoto et al., 2017; Shim et al., 2018). IL-1β, IL-6, IL-18, and TNF promote Th17 cell differentiation, reduce the synthesis of cartilage components (Alippe and Mbalaviele 2019), and inhibit osteoblast generation *via* several mechanisms (Shim et al., 2018).

After encountering APCs, antigens, and other microenvironmental variables, a native CD4 T cell’s trip to becoming an antigen-specific Th17 cell may be separated into three terrains. During the first terrain, TCR–pMHC interaction strength, costimulation strength, and other “non-cytokine"-induced variables work with APC-generated cytokines (e.g., IL-6) to trigger activation of STAT proteins (e.g., STAT3, STAT1) and “pioneer” TFs such as BATF and IRF4. STAT proteins and pioneer transcription factors (TFs) begin lineage-specific development by inducing STAT-responsive and IRF4/BATF-responsive genes, including RORt. STAT3-induced RORt works with “pioneer” TFs to epigenetically modify important Th17-related genes to make them transcriptionally permissive. In its final passage through the third terrain, an orchestration of complex signaling events modulated by RORt along with lineage-associated TFs (e.g., Runx1, AhR, and c-Maf) determines the stability of the Th17 developmental program by integrating pro-inflammatory and anti-inflammatory environmental cues. Interplay of varied elements across three terrains determines Th17 cell plasticity (Bhaumik and Basu 2017). Autoimmunity to a number of self-proteins has been linked to the onset and development of RA. Some of the antigens described come from joints, like type II collagen and human cartilage-derived glycoprotein HCgp39 (Tsark et al., 2002). Other antigens are stress-associated proteins, like grp78/BiP, which is an intracellular chaperone involved in endoplasmic reticulum stress and angiogenesis in proliferating RA synovial tissue (Bläss et al., 2001; Yoo et al., 2012). Endoplasmic reticulum stress can be caused by many things, such as proinflammatory cytokines, cell death, proteins in the endoplasmic reticulum that do not fold correctly, and reactive oxygen species (Hasnain et al., 2012). Because of the flow of Ca2+ inside the cell, the peptidyl arginine deiminase enzymes are turned on, which speeds up the deamination of arginine to citrullination (Anzilotti et al., 2010). The function of NLRP3 in mediating DC-dependent Th17 cell responses has also been seen in *Bordetella* *pertussis* and *Candida* *albicans* infection models (Dunne et al., 2010; van de Veerdonk et al., 2011). NLRP3 hyperactivation, on the other hand, generates increased IL-1 synthesis from APCs, resulting in enhanced Th17 cell differentiation and Th17 cell-dominant immunopathology (Meng et al., 2009). In autoimmune, infectious, and inflammatory scenarios, activation of the NLPR3-mediated inflammasome by DCs promotes Th17 cell development through IL-1 and/or IL-18 (Huang et al., 2012). In addition, when the inflammasome is turned on in murine macrophages, citrullination is caused (Mishra et al., 2019).

Tofacitinib (TOF) is a Janus kinase (JAK) inhibitor that ameliorated joint inflammation and damage in a collagen-induced arthritis (CIA) model by suppressing γδT17 cell activation mediated *via* inhibition of the NLRP3 inflammasome (Yang et al., 2021), which shows the relationship between NLRP3 and Th17 cells. Osteoblasts and osteoclasts precisely control bone tissue, which are controlled by growth factors and hormones (Siddiqui and Partridge 2016). Macrophages, monocytes, neutrophils, and Th17 cells in bone tissue are also involved in homeostasis regulation *via* signal exchange with these cells (Sato et al., 2006; Chen et al., 2020). Increased inflammasome activity in these cells may change the functions of osteoblasts and osteoclasts to change bone mass and bone quality. During osteolysis by osteoclast activity, factors derived from the bone matrix act as DAMPs to further promote the inflammatory response and bone loss. Conversely, inhibition of bone resorption reduces the inflammasome activity of bone constituent cells (Alippe et al., 2017; Madel et al., 2019). Therefore, inflammasome activation is a cellular action that promotes bone resorption but may also be the result of inflammatory bone loss. These findings show that inflammasome activation plays a positive feedback role in inflammatory bone loss. NLRP3 inflammasome activity increases bone resorption activity in osteoclast lineage cells and promotes IL-1β and IL-18 production. The increase in pathogen-associated molecular patterns (PAMPs) and DAMPs involved in osteolysis promotes polynuclearization and IL-1β and IL-18 production and increases inflammasome activity in osteoclasts and osteoclast progenitors (Kim H. W et al., 2017). IL-1β induces proliferation and multinucleation of osteoclasts derived from hematopoietic lineage cells in the presence of macrophage colony-stimulating factor (M-CSF) and receptor activator of nuclear factor kappa B ligand (RANKL) (Cao et al., 2016). However, it also induces the migration of osteoclast precursors, which results in quantitative changes in M-CSF, osteoprotegerin (OPG), and C-X3-C motif ligand 1 (CX3CL1) involved in hyperdifferentiation (Roper et al., 2020).

**FIGURE 2:**
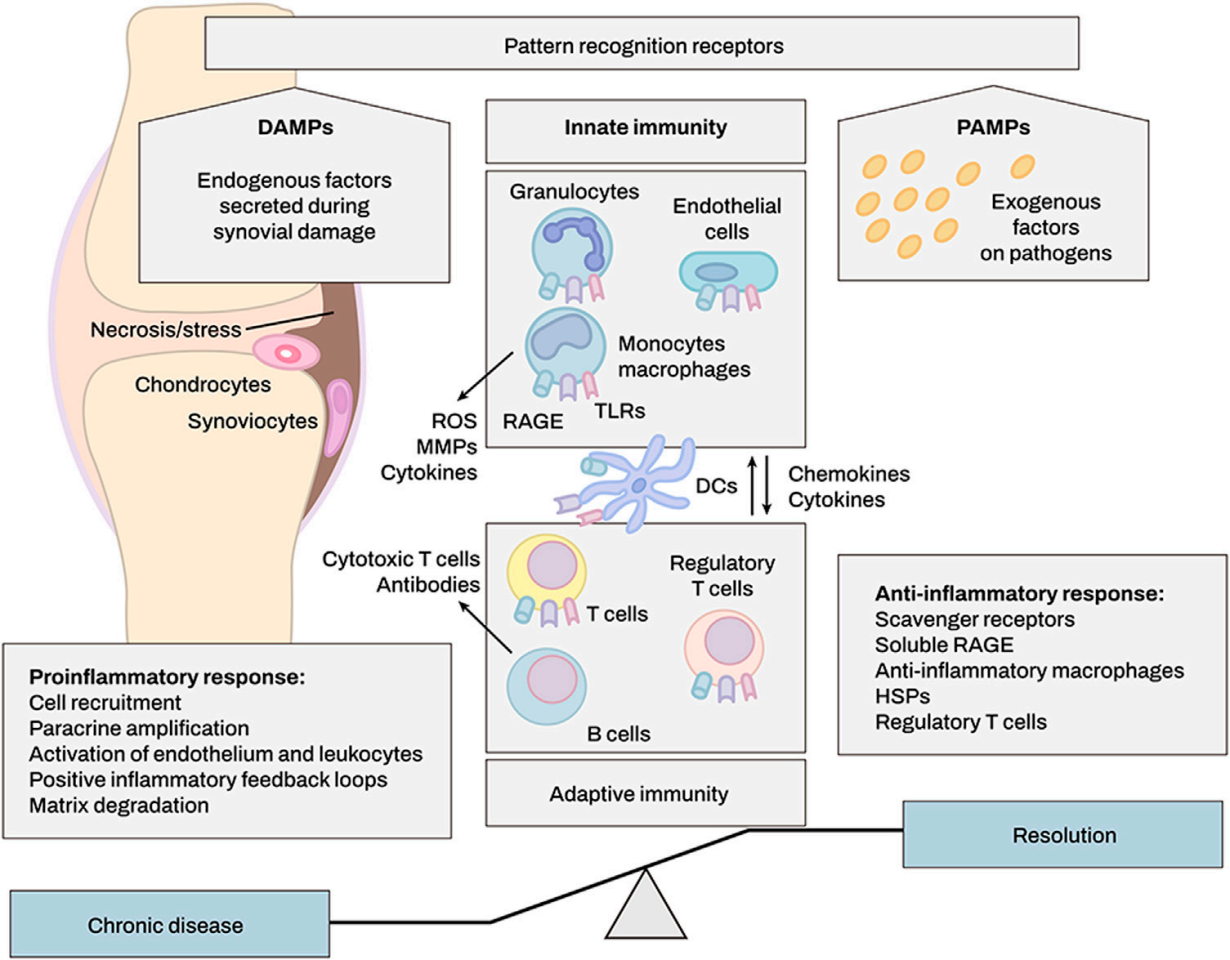
DAMPs produced upon synovial tissue damage regulate immune responses. DAMP molecules activate immune cells after cell injury, infection, or inflammation. Innate immune system may enhance and alleviate inflammation by eliminating or modulating adaptive immune responses, e.g., through regulatory T cells. Positive feedback loops involving DAMPs increase leukocyte recruitment and cause persistent rheumatoid arthritis.

DAMPs are classified as endogenous stimuli produced by dying cells or extracellular matrix (ECM) (Frevert et al., 2018). Internal cellular components such as heat shock proteins and the extracellular matrix (ECM) are common sources of DAMPs (e.g., biglycan, decorin). They are released into the extracellular milieu upon tissue damage, where they may either exacerbate inflammation or promote tissue regeneration through activation of pattern recognition receptors (PRRs) (Figure 2) (Frevert et al., 2018). The biological activity of these DAMPs is dependent on the activation of PRRs including Toll-like receptors (TLRs), NOD-like receptors (NLRs), and Receptor for Advanced Glycosylation End products (RAGEs) (Barreto et al., 2015). Barreto et al. also demonstrated that soluble biglycan is commonly detected in knee synovial fluid of patients with advanced knee OA or rheumatoid arthritis (RA) (Barreto et al., 2015).

*In vivo* investigations have shown that the NLRP3 inflammasome promotes bone resorption under estrogen deprivation and prolonged parathyroid hormone exposure, and that NLRP3 deletion lowers bone loss in numerous high bone turnover scenarios (Alippe et al., 2017). In osteoclasts exposed to high glucose concentrations in the rat model of diabetic osteoporosis, NLRP3 inflammasome and associated proteins are upregulated, bone density is decreased, and osteoclast markers are upregulated (Zhang et al., 2021). MSC exosomes may suppress the overactive NLRP3 inflammasome (Zhang et al., 2021). The NF-κB pathway initiates NLRP3 inflammasome activation. NF-κB regulates inflammation downstream of TLRs and the immune response’s traditional inflammatory pathways. NF-κB pathway affects formation and maturation of osteoblasts and osteoclasts, according to many studies (Novack 2011). High glucose upregulates NLRP3 inflammasome expression, which is controlled by ROS/MAPKs/NF-κB. NF-κB inhibitors diminish NLRP3 expression and bone resorption (An et al., 2019). The NLRP3 inflammasome and the conventional inflammatory pathway mutually conflict and affect osteoclast development, confirming NF-κB’s function in initiating the NLRP3 inflammasome (Jiang et al., 2021). IL-1β suppresses osteogenic differentiation by dampening the BMP/Smad pathway and other osteogenic markers such RUNX2, OCN, and ALP. IL-1β promotes activated osteoclasts *via* a RANKL-RANK independent pathway after binding with IL-1R on T cells or B cells and inducing the production of RANKL on osteoblasts (Jiang et al., 2021).

## Ankylosing spondylitis and the inflammasome

The initial stage of AS is inflammation, and controlling inflammation may be the key to finding ways to prevent other major symptoms of AS. is Inflammasome activation is a hallmark of an inflammatory response mediated primarily by macrophages (Yi 2018). Although few studies investigated the relationship between AS and the NLRP3 inflammasome, there is accumulating evidence of the constituents of the NLRP3 inflammasome or related expression factors in AS pathology. Levels of caspase-1 in inflammasomes were higher in AS than other types of arthritis (Kim et al., 2022). High *NLRP3* and *ASC* gene expression and a correlation between AS and the NLRP3 inflammasome were reported (Kim et al., 2018). However, another study observed intestinal overexpression of NLRs and AIM2 in patients with AS and suggested a link between the microbiome and inflammasome activation (Guggino et al., 2021). Anakinra, which inhibits IL-1, is effective in treating AS (Tan et al., 2004; Yi 2018). MicroRNA (miR)-21 expression in response to JAK2/STAT3 signaling activation by TNF-α exposure caused osteogenesis in AS (Zou et al., 2020), which suggests a correlation between the NLRP3 inflammasome and AS (Zhu et al., 2021). There is an association between various inflammatory cytokines and AS related to the NLRP3 inflammasome (Zeng et al., 2011; Yan et al., 2014; Maxwell et al., 2015). However, the relationship between AS and the NLRP3 inflammasome has been revealed indirectly, and further research is needed to determine the direct correlation between AS and the NLRP3 inflammasome.

## Gout and the inflammasome

**FIGURE 3:**
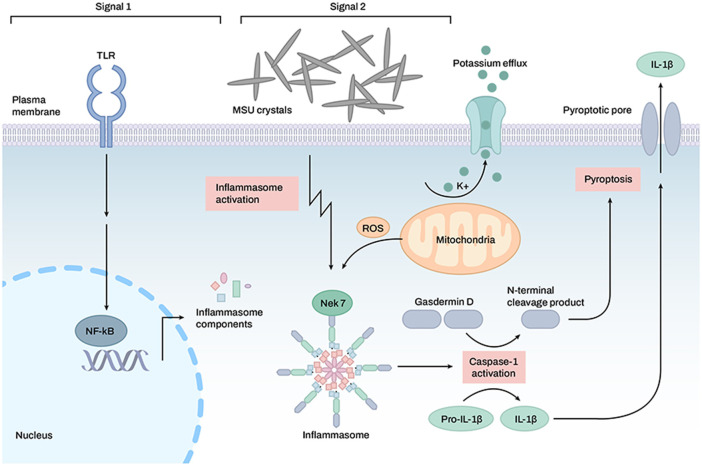
Activation of the inflammasome by monosodium uric acid crystals in the induction of gouty arthritis symptoms. TLR receptors are activated, and inflammasome formation is mediated by monosodium uric acid crystals. Once the inflammasome is activated, caspase-1 is also activated, which allows for the cleavage of GSDMD, pro-IL-1β, and pro-IL-18. Active IL-1 and IL-18 may be exported to the extracellular space *via* a transmembrane channel formed by the N-terminal GSDMD.

The NLRP3 inflammasome plays a pivotal role in the development of acute gout because the MSU crystal is an agonist of the NLRP3 inflammasome (Martinon et al., 2006). The activated NLRP3 inflammasome degrades pro-IL-1β and pro-IL-18 into mature IL-1β and IL-18, respectively (Figure 3) (Dinarello 1998; Sutterwala et al., 2006). Increased IL-1β production is a critical pathogenic hallmark of gouty inflammation and attack.

Uric acid is released from damaged cells and acts as a DAMP to activate the NLRP3 inflammasome (Shi et al., 2003; Martinon et al., 2006). Once uric acid crystals are phagocytosed by macrophages, phagosomes are ruptured by crystals, and phagosomal contents, such as cathepsins, proteases, and Ca^2+^, are released into the cytosol (Hornung et al., 2008). Uric acid crystal-induced activation of the NLRP3 inflammasome is accompanied by mitochondrial ROS generation and K+ efflux (So and Martinon 2017). Uric acid crystals induce the binding of TXNIP with NLRP3, which may occur *via* the production of ROS, and initiate NLRP3 inflammasome assembly (Kim et al., 2019).

The critical roles of the NLRP3 inflammasome and IL-1β in gout pathology have been revealed, and a blocking strategy for these pathways has emerged as a promising new therapeutic for gout attack. NLRP3 inflammasome inhibitors and IL-1 inhibitors, such as anakinra (an IL-1R antagonist), rilonacept (IL-1 TRAP), and canakinumab (monoclonal anti-IL-1β antibody), have been tested in clinical trials and showed efficacy in the treatment of acute and chronic gout patients.

## Therapeutic approaches

In the acute phase response, NSAIDs (naproxen, ibuprofen, coxibs) are used to decrease pain by reducing inflammation. NSAIDs exert their pharmacological action by inhibiting cyclooxygenase (COX), particularly inflammation-induced COX-2. However, the potential of injury must be addressed, since suppression of prostaglandins may result in major adverse effects, such as bleeding, gastrointestinal ulcers, renal failure, heart failure, rashes, disorientation, confusion, seizures, and more. Using COX-2-selective NSAIDs (celecoxib, rofecoxib, valdecoxib) may help prevent some of the negative effects (Crofford 2013). It has been known that rheumatoid arthritis may be effectively treated by inhibiting cytokines. Anakinra is a recombinant human IL-1 receptor antagonist that inhibits IL-1α and IL-1β competitively (Schwaid and Spencer 2021). Although the Food and Drug Administration (FDA) has licensed Anakinra for the treatment of rheumatoid arthritis in certain individuals (Mertens and Singh 2009), it is only moderately effective and inferior to TNF-α inhibitors, showing its limited application (Ramírez and Cañete 2018). Additionally, canakinumab, a human monoclonal IL-1β antibody, and rilonacept, a decoy receptor of IL-1α and IL-1β, have been targeted in rheumatoid arthritis (Swanson et al., 2019).

**FIGURE 4:**
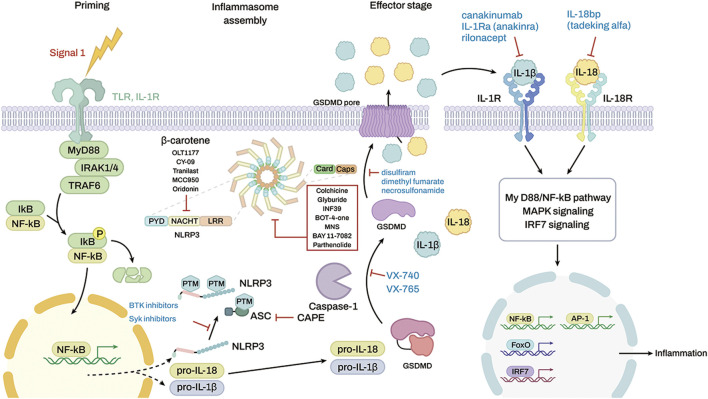
Targeting inflammasomes by inhibitors and the molecular targets of some anti-inflammatory drugs. The red box shows a list of inhibitors that stop the inflammasome function, but the molecular targets are unknown.

Many compounds inhibit the NLRP3 inflammasome (Figure 4) and show potential therapeutic efficacy for rheumatic diseases (Table 1). MCC950 inhibits AIM2, NLRC4, and NLRP1 activation in mouse macrophages, human monocyte–derived macrophages, human PBMCs, and canonical and noncanonical NLRP3 activation (Coll et al., 2015). MCC950 treatment effectively attenuated the inflammatory symptoms of NLRP3-related diseases, including experimental autoimmune meningitis and cryopyrin-associated periodic syndrome (CAPS) (Coll et al., 2015). OLT1177 is composed of β-sulfonyl nitrile that inhibits canonical and noncanonical NLRP3 activation (Marchetti et al., 2018a). OLT1177 lowers caspase-1 activity and IL-1 production in monocytes from patients with CAPS, limits adenosine triphosphatase (ATPase) activity, and binds directly to the NLRP3 protein. It also reduces LPS-induced systemic inflammation in rats (Marchetti et al., 2018b). The NLRP3 inflammasome inhibitors BAY 11-7082, BOT-4-one, parthenolide, INF 39, NSC 170724, and OLT1177 have median inhibitory concentration (IC50) values between 1 and 10 μM. However, this effect does not lead to a direct and specific binding action to NLRP3. The off-target effects cause BAY 11-7082, BOT-4-one, parthenolide, and INF 39 to exhibit a variety of behaviors (Jiang et al., 2017). Because the biological activity of NSC 170724 and OLT1177 in an NLRP3-dependent model could not be assessed, the real mechanism of inhibition is not clear. Oridonin creates a covalent link with NLRP3 while simultaneously inhibiting the activity of NF-κB and mitogen-activated protein kinase (MAPK) (He et al., 2018). Although CY-09 was designed as a derivative of a cystic fibrosis transmembrane conductance regulator (CFTR) inhibitor, a subsequent study showed that CY-09 preferentially bound to the NACHT ATPase site of NLRP3. IL-1β has an IC50 value of 5 μM (Jiang et al., 2017). β-Carotene inhibits macrophage activation by ATP, MSU crystals, and nigericin. β-Carotene reduced inflammation in a mouse model of gouty arthritis caused by MSU crystals (Yang et al., 2020a) and reduced IL-1β release in human synovial cells from patients with gout, which suggest an antigout action (Yang et al., 2020b). So far, three firms have entered the clinic with NLRP3 inhibitors (Schwaid and Spencer 2021). Olatec has published phase II results in acute gout for their chemical dapansutrile. This is the most clinically progressed NLRP3 inhibitor at the present (Jansen et al., 2019). Dapansutrile reduced joint discomfort by more than 50% after 3 days of therapy in their trial. Dapansutrile was given in doses of 100, 300, 1,000, or 2000 mg per day. Notably, there was no improvement in effectiveness beyond the 300 mg/day level, and treatment responses were identical in all groups after 3 days. Pain relief ranged from 56 to 68% on day 3 in individuals treated with 300 mg/day, which is comparable to pain relief found with NSAIDs or prednisolone (Klück et al., 2020).

**TABLE 1:** Inhibitors of the NLRP3 inflammasome in rheumatic diseases.

Agents	Diseases	Targets	Experimental models	References
Anakinra	Gout, rheumatoid arthritis, ankylosing spondylitis	IL-1α and IL-1β	Open-labeled studies with patients	Tan et al. (2004)
Auranofin	Rheumatoid arthritis	IKK kinase, thioredoxin reductase	Macrophages, human sebocytes	(Isakov et al., 2014; Yang et al., 2020b)
β-carotene	Gout	NLRP3 PYD domain	Surface plasmon resonance analysis with a recombinant protein, mouse gouty arthritis models, primary synovial fluid cells from gout patients	Yang et al. (2020a)
Caffeic acid phenethyl ester	Gout	ASC	Surface plasmon resonance analysis with a recombinant protein, mouse gouty arthritis models	Lee et al. (2016)
Canakinumab	Gout	IL-1β	A double-blind, randomized study, A 12-week, Phase II, dose-finding study	Alten et al. (2011)
	Rheumatoid arthritis			
CY-09	Gout	NLRP3 NACHT domain (Walker A motif)	Synovial fluid cells from patients with gout	Jiang et al. (2017)
EGCG	Gout	Mitochondrial DNA	Primary macrophages and an acute gout mouse model	Lee et al. (2019)
β-hydroxybutyrate	Urate crystal–induced peritonitis	K+ efflux, ASC aggregation	A mouse model of urate crystal–induced peritonitis	Youm et al. (2015)
OLT1177	Degenerative arthritis	NLRP3 NACHT domain (ATPase region)	Monocytes isolated from patients with cryopyrin-associated periodic syndrome, murine acute arthritis models	Marchetti et al. (2018a)
Oridonin	Gout	NLRP3 NACHT domain (cysteine 279)	A gouty arthritis mouse model	He et al. (2018)
Rilonacept	Gout	IL-1	A randomized, controlled clinical trial	Terkeltaub et al. (2013)
Sulforaphane	Gout	AMP-activated protein kinase/autophagy axis	An acute gout mouse model	Yang et al. (2018)
Tranilast	Gout	NLRP3 NACHT domain	A gouty arthritis mouse model and synovial fluid mononuclear cells from gout patients	Huang Y et al. (2018)
VX-740, VX-765	Osteoarthritis	Caspase-1	Murine models of osteoarthritis	Cornelis et al. (2007)

Small endogenous non-coding RNAs called microRNAs (miRNAs) play a role in the post-transcriptional control of gene expression. They have a length of around 22 nucleotides. A growing body of research has shown that miRNAs are crucial in the development of certain autoimmune illnesses, such as RA, systemic lupus erythematosus (Xie and Xu 2018; Senousy et al., 2019), Sjögren’s syndrome (Jang et al., 2019), and systemic sclerosis (Iwamoto et al., 2016). Several microRNAs (miRNAs) post-transcriptionally regulate NLRP3 expression in RA. Reduced levels of miR-20a (Jin et al., 2021) and miR-21 (Dong et al., 2014; Guggino et al., 2018) aggravate RA by triggering the NLRP3 inflammasome pathway and boosting STAT3 expression, while lowering STAT5 expression, both of which are linked with an imbalance of Th17/Treg cells (Huang Q et al., 2018). MiR-223 is a negative regulator of NLRP3, and miR-223–3p targets the 3′-UTR of NLRP3 to reduce its expression in fibroblast-like synoviocytes (Bauernfeind et al., 2012; Wu et al., 2020). MiR-30a reduces the expression of NLRP3 in synovial macrophages *via* direct binding to the 3ʹ UTR, and AAV-miR-30a injection inhibited NLRP3 inflammasome activation in mice. A20 is a de-ubiquitinase and an E3 ligase that is induced by inflammatory cytokines, and it acts as a negative regulator of NF-κB signaling in the TLR and TNF-α pathways. Single nucleotide polymorphisms (SNPs) of A20 are associated with high susceptibility to autoimmune diseases and rheumatoid arthritis. Lack of A20 in myeloid cells initiated a spontaneous polyarthritis in mice and enhanced the expression levels of NLRP3 and pro-IL-1β in macrophages (Vande Walle et al., 2014). Deficiency of NLRP3 or caspase-1/-11 in A20 knockout mice protected against the inflammatory symptoms of rheumatoid arthritis, which showed that A20 negatively regulated NLRP3 inflammasome activity in rheumatoid arthritis (Vande Walle et al., 2014). These results suggest that intracellular signaling targets regulate NLRP3 inflammasome activity and directly target inflammasome components. The recent research suggests that miRNAs are promising candidates for better understanding the pathogenetic processes of RA and designing more effective treatment strategies.

Although there is currently no clinically accepted treatment that targets or modulates NLRP3 inflammasome activation, these investigational compounds may regulate its anti-inflammatory action in arthritic disease *via* several signaling pathways.

## Conclusion and perspective

The role of the NLRP3 inflammasome in autoinflammatory disorders has been established. Patients with arthritis benefit enormously from the introduction of inflammasome-targeted biologics. Research on the activation of diverse inflammasomes has expanded, and the results have allowed for the development of highly targeted and effective inhibitors. Human cell models, rather than animal models, are required for the successful translation of newly identified inflammasome inhibitors into (pre)clinical trials. Differences between human and murine inflammasome biology alter the treatment effectiveness results drawn from murine illness models. The development of appropriate cellular models harboring patient mutations and the direct use of patient-derived cells may help identify therapeutically useful medicines more quickly and reliably.
